# Gastrointestinal and urinary tract pathogenic infections among HIV seropositive patients at the Komfo Anokye Teaching Hospital in Ghana

**DOI:** 10.1186/1756-0500-5-454

**Published:** 2012-08-21

**Authors:** Yaw Agyekum Boaitey, Bernard Nkrumah, Ali Idriss, Samuel Crowther Kofi Tay

**Affiliations:** 1 Microbiology Department, Komfo Anokye Teaching Hospital, Kumasi, Ghana; 2 Kumasi Centre for Collaborative Research in Tropical Medicine, Kumasi, Ghana; 3 Malaria Research Centre, Agogo, Asante Akim North, Ghana; 4 Microbiology Department, Kwame Nkrumah University of Science and Technology, Kumasi, Ghana

**Keywords:** HIV/AIDS, *Giardia lamblia*, *Cryptosporidium*, Parasitic infection

## Abstract

**Background:**

Gastrointestinal and urinary tract pathogenic infections are aggravating the incidence and progression of the Human Immunodeficiency Virus (HIV) infection into Acquired Immune Deficiency Syndrome (AIDS) more especially in the developing countries. This study was conducted to assess the common gastrointestinal and urinary infections among HIV/AIDS patients at the Komfo Anokye Teaching Hospital (KATH) in Ghana between April and December 2008.

**Findings:**

This work reports on gastrointestinal and urinary tract pathogenic infections among 500 HIV seropositive and 300 HIV seronegative patients. There was a 35% (175/500) prevalence of intestinal parasites among HIV seropositive patients compared to 4.3% (13/300) in HIV seronegative patients. *Giardia lamblia* and *Cryptosporidium* accounted for 19% (95/500) and 14% (70/500) respectively, while *Schistosoma mansoni*, *Strongyloides stercoralis* and hookworm together accounted for 2% (10/500) of intestinal parasitic infections among the HIV seropositive patients. There was no significant difference (p > 0.05) in urinary parasitic infection between HIV seropositive 1% (2/500) and seronegative patients 0.7% (2/300). Most, 60 (86%) out of 70, of the urinary tract infection among the HIV seropositive patients was due to bacteria with E. coli being the most predominant isolate, 28 (47%) out of 60. There was no significant difference in infections based on age and gender.

**Conclusion:**

*G. lamblia* and *Cryptosporidium* were the most common gastrointestinal parasites detected while bacteria accounted for majority of the urinary tract infections among the HIV seropositive patients at the hospital.

## Background

Parasitic infections caused by helminth and protozoans are among the most prevalent infections in humans worldwide especially, in the tropical and sub tropical areas of the developing world. The problems posed by parasitic infections have long been recognized and the subject has thus merited increasing importance over the years [1]. Several outbreaks of diarrheal diseases caused by these parasites have been reported during the last decade [2,3] and are estimated to cause over one million deaths each year [4]. Epidemiological surveys have shown that poor sanitation and inappropriate environmental conditions coupled with indiscriminate defecation, geophagy and contamination of water bodies in these countries are the most important predisposing factors [5]. Studies have shown higher rates of parasitic infections in HIV/AIDS positive than negative subjects [6]. HIV/AIDS compromises the immune system of the body thus patients with HIV/AIDS are threatened by a great number of diseases such as those caused by different kinds of biological agents including bacterial, fungal, viral, protozoa and helminth parasitic pathogens. In such patients, opportunistic parasitic gastrointestinal infections presents in the form of severe diarrhea which profoundly compromises the absorptive function of the small intestine, leading to significant mortality [7]. Helminth parasitic infections also cause chronic immune activation [8-10] which has been shown to increase host susceptibility, thereby promoting HIV/AIDS infection and disease progression [11,12]. Infections with the major human gastrointestinal parasites have been shown to be rampant in most parts of Africa and the prevalence of these infections varies from country to country [13-16] with Ghana being no exception [17]. Studies conducted in the Ashanti Region of Ghana have shown the presence of gastrointestinal infections among both children and adults [18-21]. However, information regarding the magnitude of opportunistic parasitic infections among HIV/AIDS positive individuals is scarce and not well studied. This study was therefore undertaken to contribute to the knowledge of the occurrence of gastrointestinal and urinary tract infections among HIV/AIDS positive and negative patients at Komfo Anokye Teaching Hospital (KATH) in Kumasi, Ghana.

## Methods

### Study design

This was a hospital based cross-sectional study of HIV seropositive and negative adults [22] presenting to the Voluntary Counseling and Testing Unit and Chest Clinic of the Komfo Anokye Teaching Hospital (KATH).

### Study site

The study was performed at the Voluntary Counseling and Testing Unit and Chest Clinic of KATH, Ghana from April to December 2008. KATH is approximately a thousand bed tertiary medical facility located in Kumasi, Ashanti Region, the confluence of the transportation network in the central part of Ghana. Its position makes it the most accessible tertiary and referral medical facility in Ghana attending to a population of over 4.4 million in the Ashanti region and beyond [23].

### Ethical approval

The study protocol was approved by the Committee on Human Research, Publications and Ethics, School of Medical Sciences, Kwame Nkrumah University of Science and Technology in collaboration with KATH management.

### Stool collection

The parasitology laboratory of KATH is a well established unit that operates by the internationally accepted Good Laboratory Practice (GLP). Stool samples were collected in accordance with WHO guidelines on the collection of faecal samples [24] and as outlined previously by Nkrumah et al. [20].

### Urine collection

Urine samples were collected as previously described by Cheesbrough [6].

## Specimen processing and examination

### HIV testing

HIV testing for study subjects was conducted at the VCT centre and the serology department of the hospital. Testing was done using the national algorithm. Briefly, all tests were done using the First Response HIV Card 1–2.0 (PMC Medical India Pvt Ltd). Positive tests were confirmed with OraQuick *ADVANCE* Rapid HIV-1/2 (OraSure Technologies, Inc.). Enzyme-Linked Immunosorbent Assay (ELISA) was used as a tie breaker. All tests were performed as outlined in the manufactures’ manual.

### Stool samples

#### Direct microscopy

Direct microscopy of the smear in saline (0.90% w/v NaCl solution) and Lugol’s iodine was performed for the detection of parasites (trophozoites, cysts, egg and larvae) as previously described [25]. In cases where parasite species identification was not conclusive the attendant microscopist requested a senior microscopist to confirm the results. Where no consensus was reached, the formol-ether concentration method was employed. The average time between sample collection and processing was 30 minutes.

#### Formol-ether concentration method

The formol-ether concentration method was performed and examined as previously described by the WHO [24] and Ayeh-Kumi et al. [25]. The average time between sample collection and processing was 50 minutes.

### Modified Ziehl Neelsen (ZN) staining method

The modified ZN staining method was used for the identification of *Cryptosporidium*. This method was performed and examined as earlier described by Ayeh-Kumi and his group [25].

### Urine samples

#### Microscopic examination-urine sedimentation method

Urine samples were processed and examined as described by Cheesbrough [6]. Urine samples with pus cells > 5 /HPF were cultured. The average turnaround time was 20 minutes.

#### Culture process

Urine samples were cultured using a standard loop calibrated to hold 0.01 ml of urine onto Cysteine Lactose Electrolyte Deficient (CLED) agar. Inoculated plates were incubated at 37°C aerobically overnight. After overnight incubation the plates were read and the growths were identified based on the growth characteristics on the inoculated media.

#### Colony counts

Colonies were counted on CLED and multiplied by the loop volume. A bacterial count of 1x10^5^ per ml was considered significant for UTI while counts less than 1x10^5^ per ml were considered as no significant bacterial growth [6].

#### Bacterial identification

Bacterial isolates were identified using conventional biochemical methods including urease and indole production, citrate utilization, hydrogen sulphide, gas production and fermentation of sugars. The biochemical media used included Simon’s Citrate medium, Urea and Triple Sugar Iron agar (TSI). Catalase and coagulase tests were performed for all staphylococci organisms.

#### Antimicrobial sensitivity testing

Antimicrobial susceptibility test were performed using Kirby-Bauer disc diffusion method. The isolates from this study were tested against the following antibiotics: Ampicillin (10 μg), Cefuroxime (30 μg), Cotrimoxazole (25 μg), Ciprofloxacin (5 μg), Gentamicin (10 μg), Tetracycline (30 μg), Nalidixic acid (30 μg) and Nitrofurantoin (300 μg). Zone sizes were measured according to CLSI standards. The average turnaround time was 2 days.

## Findings

A total of 800 subjects were examined of which 500 (62.5%) out of 800 were HIV seropositive subjects. Females constituted 52.7% (419/800) of the total study population. Out of 500 HIV seropositive patients, 258 (51.6%) presented with diarrhea or fever. Out of these 258, 149 (57.8%), 60 (23.3%) and 10 (9.2%) were positive for intestinal parasites, bacteruria or both respectively. Subjects within 21–30 years constituted the highest age group of the study population 38.4% (307/800) (Table 1). Out of 500 HIV seropositive patients, 365 (73%) and out of 300 HIV seronegative patients, 217 (72.3%) were recruited within the wet season respectively. There were more symptomatic seropositive subjects in the wet season; 61.4% (224/365), than in the dry season; 25.2% (34/135), with more parasites being isolated from symptomatic subjects within the wet season 87.5% (196/224).

**Table 1:** Overall infection rates by age group of the studied populations

**Age groups**	**Males**			**Females**			**Total**			**Males**			**Females**			**Total**		
	**No**	**Parasite +**	**(%)**	**No**	**Parasite +**	**(%)**	**No**	**Parasite +**	**(%)**	**No.**	**Parasite +**	**(%)**	**No**	**Parasite +**	**(%)**	**No**	**Parasite +**	**(%)**
≤ 20	23	4	17.3	26	6	23	49	10	20.4	23	0	0	26	0	0	49	0	0
21-30	152	42	27.6	155	40	25.8	307	82	26.7	152	2	1.3	155	1	0.6	307	3	0.9
31-40	144	30	20.8	154	37	24.2	298	67	22.5	144	1	0.7	154	2	1.2	298	3	1
41-50	60	11	18.3	70	15	21.4	130	26	20	60	0	0	70	1	1.4	130	1	0.8
> 50	2	-	0	14	3	21.4	16	3	18.8	2	0	0	14	0	0	16	0	0
Total	381	87	22.8	419	101	24.1	800	188	23.5	381	3	0.8	419	4	0.9	800	7	0.9

Gastrointestinal infection rate Urinary parasitic infection rate.

Gastrointestinal infection was found in 175 (35%) out of 500 HIV seropositive subjects, and only in 13 (4.3%) out of 300 HIV seronegative subjects, with a statistically significant difference (p < 0.05). However, there was no significant difference in urinary infection among 5 (1.0%) out of 500 HIV seropositive subjects and 2 (0.7%) out of 300 seronegative subjects (p > 0.05) (Table 2). Among the various age groups, there was no significant difference (p > 0.05) in urinary parasitic infection rate. Infection with *G. lamblia* (19%) and *Cryptosporidium* (14%) were statistically higher in HIV seropositive subjects (p < 0.05) (Table 3). Out of 70 HIV seropositive patients suspected of urinary tract infection, 86% had bacteruria while 28% was recorded for HIV seronegative patients (n = 18).

**Table 2:** Overall parasite infection according to sex of the studied populations

**Patient groups**	**Males**			**Females**			**Total**				**Males**			**Females**			**Total**	
	**No.**	**Parasite +**	**(%)**	**No.**	**Parasite +**	**(%)**	**No.**	**Parasite +**	**(%)**	**No.**	**Parasite +**	**(%)**	**No.**	**Parasite +**	**(%)**	**No.**	**Parasite +**	**(%)**
HIV + patients	241	82	34	259	93	35.9	500	175	35	241	2	0.8	259	3	1.1	500	5	1
HIV - patients	140	5	3.6	160	8	5	300	13	4.3	140	1	0.7	160	1	0.6	300	2	0.7
Total	381	87	22.8	419	101	24.1	800	188	23.5	381	3	0.8	419	4	0.9	800	7	0.9

Gastrointestinal parasitic infection.

Urinary parasitic infection.

**Table 3:** Gastrointestinal parasites among HIV seropositive and negative subjects

**Parasites**	**HIV positive (n = 500)**	**%**	**HIV negative (n = 300)**	**%**	**Total (n = 800)**	**%**
*Schistosoma masoni*	3	0.6	2	0.7	5	0.6
*Giardia lamblia*	95	19	6	2	101	12.6
*Stronglyloides stercolaris*	2	0.4	1	0.3	3	0.4
Hookworm	5	1	2	0.7	7	0.9
*Cryptosporidium* sp.	70	14	2	0.7	72	9
Total	175	35	13	4.3	188	23.5

One of the most important clinical manifestations in HIV/AIDS patients is diarrhea. This might be caused by various etiological agents including parasitic coccidian and flagellates, bacteria and fungi. Most of these agents can cause life threatening chronic watery diarrhea, weight loss and malabsorption. This makes the persons’ sense of well being and ability to carry out daily activities profoundly impaired [26]. A prevalence of 35% of intestinal parasites among the HIV seropositive patients in this study corresponds with reports from other African countries. Awole et al. [16] reported a prevalence rate of 44.8% among HIV infected patients in Ethiopia while Agi [4] recorded 33.6% in Sagbanna community of Niger-Delta in Nigeria. These high prevalence rates are consistent with the fact that, one of the factors that predispose people to parasitic infection is the presence of co-existing disease (example HIV infection) or other conditions which reduces natural immune responses [6]. The low urinary infection rate (1.0%) and the high prevalence of bacterial infection (86%) among the HIV seropositive patients suggest that suspected urinary tract infection (urine samples with pus cells > 5/HPF) among these patients are mostly due to bacterial infections but not of parasitic source. E coli and Staph aureus were the most isolated organisms. The antibiotic sensitivity patterns from this study showed that most of the *E. coli* isolated was sensitive to nitrofurantoin, gentamicin, cefuroxime and ciprofloxacin. This sensitivity pattern is consistent with a study earlier conducted by Turpin and his colleagues within the same hospital [27] and also consistent to results obtained by Obirikorang and friends in another study conducted within the study area [28]. This low prevalence of urinary parasitic infection is consistent with a 6 year retrospective and prospective study of urinary parasitic infections at KATH which also found that these parasites are less commonly found in routine urine analysis (unpublished data). The even distribution of the parasitic infections in both sexes as well as, among the various age groups suggested that sex and age were not predetermining factors for parasitic infections. The result of this study also revealed a trend in the occurrence of specific intestinal parasites in HIV seropositive patients: *G. lamblia* (19%), *Cryptosporidium* (14%), *Hookworm* (1%), *Schistosoma mansoni* (0.6%) and *Strongyloides stercoralis* (0.4%). The results of this study disagreed with those of Lindo et al. [29] who found *Trichuris trichuria* (21.1%), Hookworm (17.3%) and *Strongyloides stercoralis* (7.7%) in stool samples of HIV infected individuals in San Pedro Sula, Hunduras in Central America. Makri et al. [30] observed that AIDS patients made up a substantial proportion of reported cryptosporidiosis cases. The high prevalence of *Cryptosporidium* in this study confirmed this parasite as one of the usual opportunistic pathogens commonly associated with HIV infection [6] even though a previous study at the University of Ghana Medical School reported the prevalence of *Cryptosporidium* species in both HIV seropositive and seronegative individuals with no significant statistical association between cryptosporidiosis and HIV infection [7]. *S. stercoralis* prevalence (0.4%) among the HIV seropositive patients in this study was not significant but very important as it can assume a role of extreme gravity due to the immune state of these individuals. This is because this parasitic helminth matures very fast and is capable of autoinfection; the larvae can easily cross the blood brain barrier and then invade the brain tissues [31] which can be very fatal. Apart from *S. stercoralis*, low prevalence rates were recorded for hookworm (1%) and *S. mansoni* (0.6%). These low rates could be as a result of three main reasons: (1) the effective counseling and continuous education on hygienic practices for HIV seropositive individuals, (2) the high prevalence of *G. lamblia* and *Cryptosporidium* sp. compared to hookworm and *S. mansoni* among HIV/AIDS patients within the study area, (3) the complex mode of infection of hookworm and *S. mansoni* compared to that of *G. lamblia* and *Cryptosporidium* sp*.* Even though the residential areas of the patients were not investigated these parasites are associated with poor environmental sanitation and lack of good water supply [5]. There was also a relationship between the seasonality, symptomatology and parasitemia.

## Conclusion

The results of the study revealed *G. lamblia* and *Cryptosporidium* as the commonest intestinal parasitic agents among HIV seropositive patients at the Komfo Anokye Teaching Hospital. Most of the urinary tract infections in these patients were due to bacteria than parasites while age and sex of patients played no role in the prevalence of these infections. Techniques such as modified Ziehl Neelsen stain should be considered for clinically suspected or symptomatic HIV/AIDS patients so that parasites such as *Cryptosporidium* which are common in these patients will not be missed.

## Abbreviations

WHO: World Health Organization; UTI: Urinary Tract Infection; HIV: Human Immunodeficient Virus; AIDS: Acquired Immune Deficiency Syndrome; KATH: Komfo Anokye Teaching Hospital; HPF: High Power Field; CLSI: Clinical and Laboratory Standards Institute; TSI: Triple Sugar Iron.

## Competing interests

The authors declare that they have no competing interest.

## Authors' contributions

YAB planned and designed the study protocol, carried out the study and contributed to the writing of the manuscript. SCKT supervised the study and contributed to the writing of the manuscript, AI contributed to the study and writing of the manuscript and BN contributed to the study and headed the writing of the manuscript. All the authors have read and approved the manuscript.
